# Identifying Abusive Head Trauma in an Infant

**DOI:** 10.7759/cureus.70245

**Published:** 2024-09-26

**Authors:** Munkana M Barthelemy, Nikita Wadhwani, Abouzahir Hind, Awatif Aheri, Ahmed Belhouss, Hicham Benyaich, Siddick Omar, Imane Guessous, Ayrton Bangolo, John Bukasa, Deepak Amin

**Affiliations:** 1 Forensic Medicine, Ibn Rochd Teaching Hospital of Casablanca, Casablanca, MAR; 2 Internal Medicine, Hackensack Meridian Health Palisades Medical Center, North Bergen, USA; 3 Pediatrics, Ibn Rochd Teaching Hospital of Casablanca, Casablanca, MAR; 4 Endocrinology, Kinshasa University Clinics, Kinshasa, COD

**Keywords:** abusive head trauma, intracranial hemorrhage, pediatric neurotrauma, retinal hemorrhage, shaken baby syndrome

## Abstract

Abusive head trauma (AHT) is a type of neurotrauma that accounts for significant morbidity and mortality. It is characterized by a constellation of neurologic and radiologic signs indicative of abuse in children aged zero to five years. Detection of these cases could be a challenging endeavor and is entirely contingent on the acumen of the healthcare professionals. It is imperative to identify suspected cases at the earliest to prevent developmental delays, visuomotor deficits, learning disabilities, and seizure disorders.

## Introduction

Abusive head trauma (AHT) results in brain injuries from excessive accelerating-decelerating forces to the head. It can result in subdural hematomas (SDHs), retinal hemorrhages, or cerebral edema. Biomechanical and clinical data have shown that shaking does not reliably reproduce these prototypical injuries. Therefore, the diagnosis is often contentious, as injuries resulting from accidental and non-accidental trauma are challenging to distinguish. Furthermore, subdural collections can result from meningitis, arachnoid cysts, and postoperative sequelae. Newer, more inclusive diagnostic terminologies, such as AHT or non-accidental trauma, have replaced older terms like shaken baby syndrome (SBS) or shaken impact syndrome [1-4]. Given the medicolegal complexities surrounding AHT, current diagnostic tools need to be improved, and awareness among the public and healthcare professionals ought to be raised to rapidly identify at-risk children and closely follow the survivors. We describe the case of an eight-month-old infant with seizures and developmental delays secondary to AHT. The requirement for informed consent was waived by the Institutional Review Board, and all data in this case report have been de-identified.

## Case presentation

An eight-month-old male infant with no previous medical history presented for evaluation of new-onset seizures. The patient reportedly experienced a spontaneously resolving episode of tonic-clonic seizure activity at home during a family gathering. There was no history of any antecedent trauma, fever, sick contacts, recent travel, or any respiratory, abdominal, or urinary complaints. Family history was non-contributory, with no history of seizures and neurological or developmental disabilities. Per the parents, the infant was up to date with vaccinations and had reached expected developmental milestones in a timely manner. He was the firstborn child in the family and had no siblings. The patient’s mother delivered the child vaginally and noted having an uneventful pregnancy course.

Vitals were stable at the time of presentation, except for a temperature of 101.2°F. No overt stigmata of physical trauma were evident on the physical exam. Complete blood count and serum chemistries were within normal limits. Initial CT of the head revealed a left epidural hematoma (EDH) along with meningeal enhancement over the left frontal region. Lumbar puncture was done to exclude meningitis. Cerebrospinal fluid (CSF) studies were within normal limits. Fundoscopic examination revealed bilateral diffuse sheet hemorrhages extending to the retinal periphery and vascular tortuosity.

**Figure 1:**
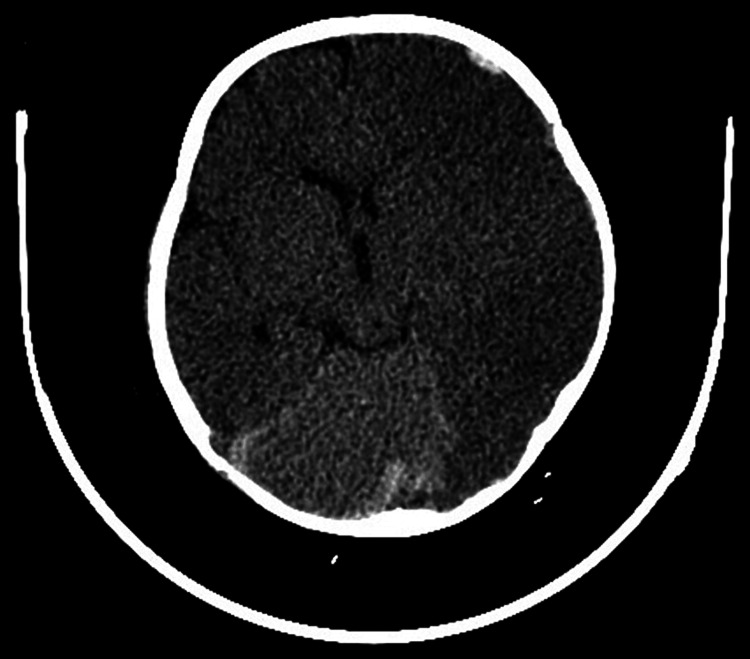
Left frontal extradural hematoma and intraparenchymal hematomas in the left occipital and right parieto-occipital regions, with juxtaparenchymal temporo-occipital hypodensities and partial effacement of the left lateral ventricle.

**Figure 2:**
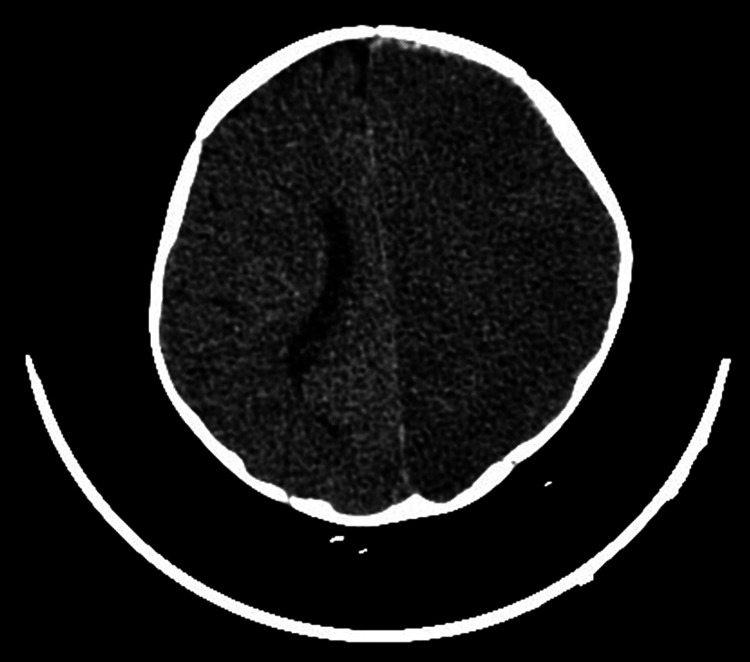
Diffuse cerebral edema involving the left hemisphere and right temporo-occipital region, with complete effacement of the left lateral ventricle.

The patient’s hospital course was complicated by the development of anisocoria, right-sided hemiparesis, and facial weakness on day 2. Repeat CT of the head showed diffuse cerebral edema involving the left hemisphere and right temporo-occipital region, effacement of the left lateral ventricle, and intraparenchymal hematoma in the left occipital and right parieto-occipital regions (Figures 1, 2). Mannitol and anti-seizure therapy were begun. Neurosurgery was consulted. The patient’s neurological deficits steadily improved with medical treatment.

Of note, some concerned family members apprised the medical team that this was the patient’s second witnessed seizure event, thereby contradicting the parents’ account of events. They also expressed concerns about the unkempt and undernourished state of the child. Further investigation into the case revealed that the mother had undiagnosed bipolar disorder and had separated from her spouse a few months prior. Additionally, she was unable to provide for the child after the recent termination of her employment and inconsistent spousal support.

Our patient required multiple corrective orthopedic procedures and orthotic braces for the correction of gait abnormalities. Despite an intensive rehabilitation program, our patient is currently wheelchair dependent due to significant motor deficits and concurrent blindness. He is on anti-seizure therapy and requires intensive caregiver support due to significant psychomotor and developmental delays.

## Discussion

AHT accounts for 53% of fatal or severe head injuries in children under two years. Etiologically, multiple mechanisms (shaking, impact, etc.), either alone or in combination, can result in traumatic injuries. Therefore, the term “abusive head trauma” is mechanistically apt and more inclusive than “shaken baby syndrome.” Subdural, intracerebral, spinal, and retinal hemorrhages, in conjunction with rib or other bony fractures that are not easily explainable by the provided mechanism of injury, should raise suspicion for AHT. Unfortunately, the diagnostic process could be confounded by conjectural and often emotionally charged courtroom arguments that are not backed by scientific literature. Evidence-based consensus guidelines need to be utilized, as AHT is a diagnosis of medical exclusion and requires meticulous scrutiny by medical experts.

The type, age, and severity of injuries, if inconsistent with the alleged mechanism, can hint at AHT. Ligamentous cervical spinal trauma appears to have a high incidence in this group. Although SDHs are most commonly seen, parenchymal insults are primarily responsible for adverse neurodevelopmental outcomes. Alternate diagnoses with similar symptomatology must be considered. Hypoxic-ischemic encephalopathy, cerebral venous sinus thrombosis, etc., may not reliably result in injuries that are prototypical of AHT.

Child abuse can result from physical, mental, and sexual trauma, or intentional caregiver negligence, with resultant grave consequences for the child’s physical and/or psychological development and well-being. Our patient was subjected to physical abuse due to untreated bipolar disorder in the mother and lack of adequate caregiver support by the father. Cranio-cerebral injuries are the leading cause of death in afflicted infants. Prodromal symptoms of SBS include unexplained uneasiness, fussiness, and occasional seizures in infants. Neurotrauma is frequently encountered in affected children and is seen more often in male infants. Discovery of an SDH or cerebral contusion in an infant on CT imaging should raise suspicion for intentional trauma [5-7]. SDHs are common due to the hydrodynamic fragility of the cortico-dural veins during infancy, especially in boys. Interestingly, our patient developed an extradural hematoma, which is seldom seen in infants. In two-thirds of cases, EDH arises due to bleeding from the middle meningeal artery during traumatic events [8].

Our patient did not show overt signs of trauma. Neuroimaging for evaluation of new-onset seizures, coupled with inconsistencies in the mother’s account of events, increased suspicion for AHT. The case was escalated to the concerned authorities, and ensuing investigation conclusively proved that the infant had endured deliberate neurotrauma.

Clinicians must painstakingly investigate cases of suspected child abuse and refer these patients to law enforcement so that a comprehensive legal investigation can be undertaken to penalize the perpetrator(s) [9]. Additionally, social workers play an indispensable role in identifying children who are being victimized by their caregivers and providing resources to streamline the rehabilitation of affected children.

## Conclusions

AHT can be a tremendous setback to a child’s psychomotor development. Symptomatology is often varied and subtle. Unexplained fussiness, bodily injuries, seizures, or neurotrauma should prompt evaluation. We incidentally discovered brain injuries in our patient during the evaluation of new-onset seizures. A formal investigation showed evidence of physical assault by the caregiver, who suffered from bipolar disorder. Child Protective Services were immediately contacted. Clinicians must escalate cases where caregiver abuse is suspected. Expeditious case recognition and multidisciplinary liaison can prove instrumental in optimizing outcomes for these children.
